# Cytomegalovirus containing TRP2 antigen provides protective immunity against the poorly immunogenic B16BL6-D5 melanoma

**DOI:** 10.1186/2051-1426-1-S1-P271

**Published:** 2013-11-07

**Authors:** Michael Neuberger, Shawn M Jensen, Guangwu Xu, Tameka Smith, Hong-Ming Hu, Ann B Hill, Carlo B Bifulco, Bernard A Fox

**Affiliations:** 1 Laboratory of Molecular and Tumor Immunology, Robert W Franz Cancer Research Center, Earle A Chiles Research Institute, Providence Cancer Center, Portland, OR, USA; 2 Laboratory of Cancer Immunobiology, Robert W Franz Cancer Research Center, Earle A Chiles Research Institute, Providence Cancer Center, Portland, OR, USA; 3 Department of Pathology, Providence Cancer Center, Portland, OR, USA; 4 Molecular Microbiology and Immunology, Oregon Health & Science University, Portland, OR, USA

## 

Cytomegalovirus is known to induce long-lived humoral and cellular immune responses that persist and increase over time. In humans on average more than 5% of T cells are CMV-reactive. We have previously reported that mouse cytomegalovirus (MCMV) encoding the unmodified TRP2 protein can induce immunity that protects mice from challenge with the weakly immunogenic B16-F10 melanoma [Xu 2013]. This protective immunity could be induced in mice with pre-existing immunity to MCMV, overcoming a critical hurdle to the clinical application of this strategy. Surprisingly, this immunity to TRP2 was mediated by antibody. We have extended these observations to the poorly immunogenic B16BL6-D5 (D5) melanoma model, where we now report that vaccination also provides protective immunity. While anti-TRP2 immunity induced by MCMV is humoral, it is as effective as vaccination with a GM-CSF-secreting (GVAX) vaccine (D5-G6) that induces tumor-specific T cell immunity. When we evaluated MCMV-TRP2 in a therapeutic model, the vaccine was ineffective as a single agent. We went on to investigate whether the "inflating" immunity that MCMV is well known for would lead to the co-development of a T cell response long-term. Mice were vaccinated with MCMV-TRP2 and followed long-term. Half of these mice were challenged with viable D5 on day 14. Controls included mice that were vaccinated with D5-G6 and followed long-term. After more than 190 days from the initial vaccination, mice were sacrificed and spleen and lymph node cells were interrogated for an IFN-γ response following stimulation with D5, murine sarcoma cells, or a peptide specific for MCMV. As expected, a large IFN-γ response was elicited by the MCMV peptide in MCMV-vector immunized mice. In addition all vaccinated animals developed a detectable IFN-γ response to the D5 melanoma. The intensity of the T cell response was similar in mice receiving MCMV-TRP2 and D5-G6. Studies are ongoing to characterize the targets of this T cell response and evaluate the mechanism(s) of MCMV-TRP2-induced tumor elimination. While preliminary, the well-established ability of CMV to induce and maintain high-frequency antigen-specific responses, combined with our results, suggest that this strategy may provide an opportunity to augment the anti-cancer immune responses.

